# Supplementary figures and images for: The neglected bee trees: European beech forests as a home for feral honey bee colonies
Source: PeerJ. 2018 Apr 6;6:e4602. doi: 10.7717/peerj.4602 (PMC5890725; doi:10.7717/peerj.4602)

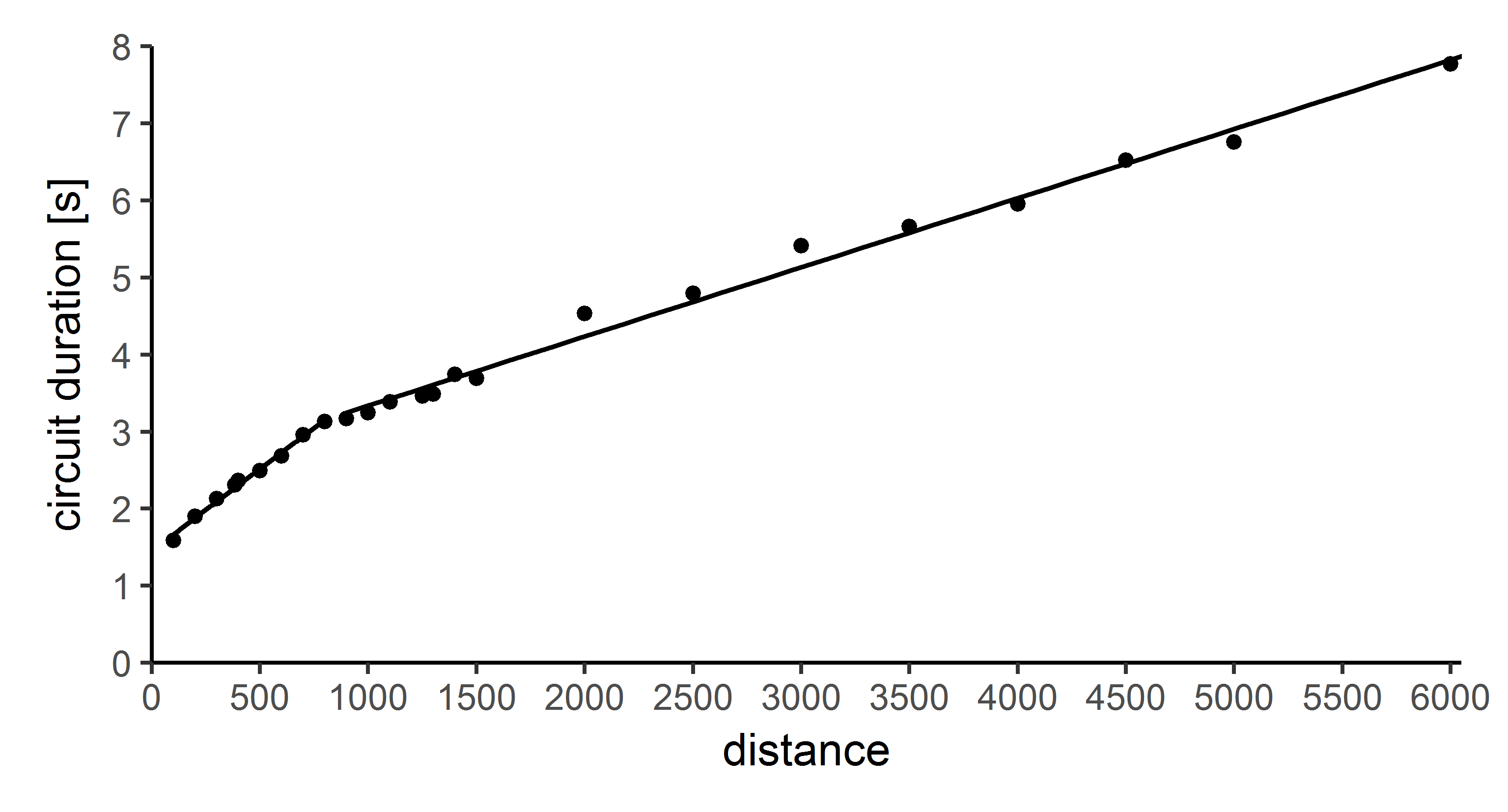

Supplement: Supplemental Information 1 — Data on dance circuit duration of waggle dancing bees in relation to flight distances as presented by von Frisch for distances between 100 and 6,000 metres. The first linear regression line well describes the relationship for distances up to about 800 metres: circuitduration (s) = 0.0021* distance (m) − 1.4552. Conversely, we used distance (m) = 466.5495* circuitduration (s) − 675.0336 to infer nest site distances from dances with circuit durations up to 3.15 seconds. The second linear regression line well describes the relationship for distances from 900 metres to 6000 metres: circuitduration (s) = 0.0009* distance (m) − 2.4391, and we used distance (m) = 1102.7328* circuitduration (s) − 2666.6256 to infer nest site distances from dances with circuit durations > 3.15 seconds. [file peerj-06-4602-s001.png]

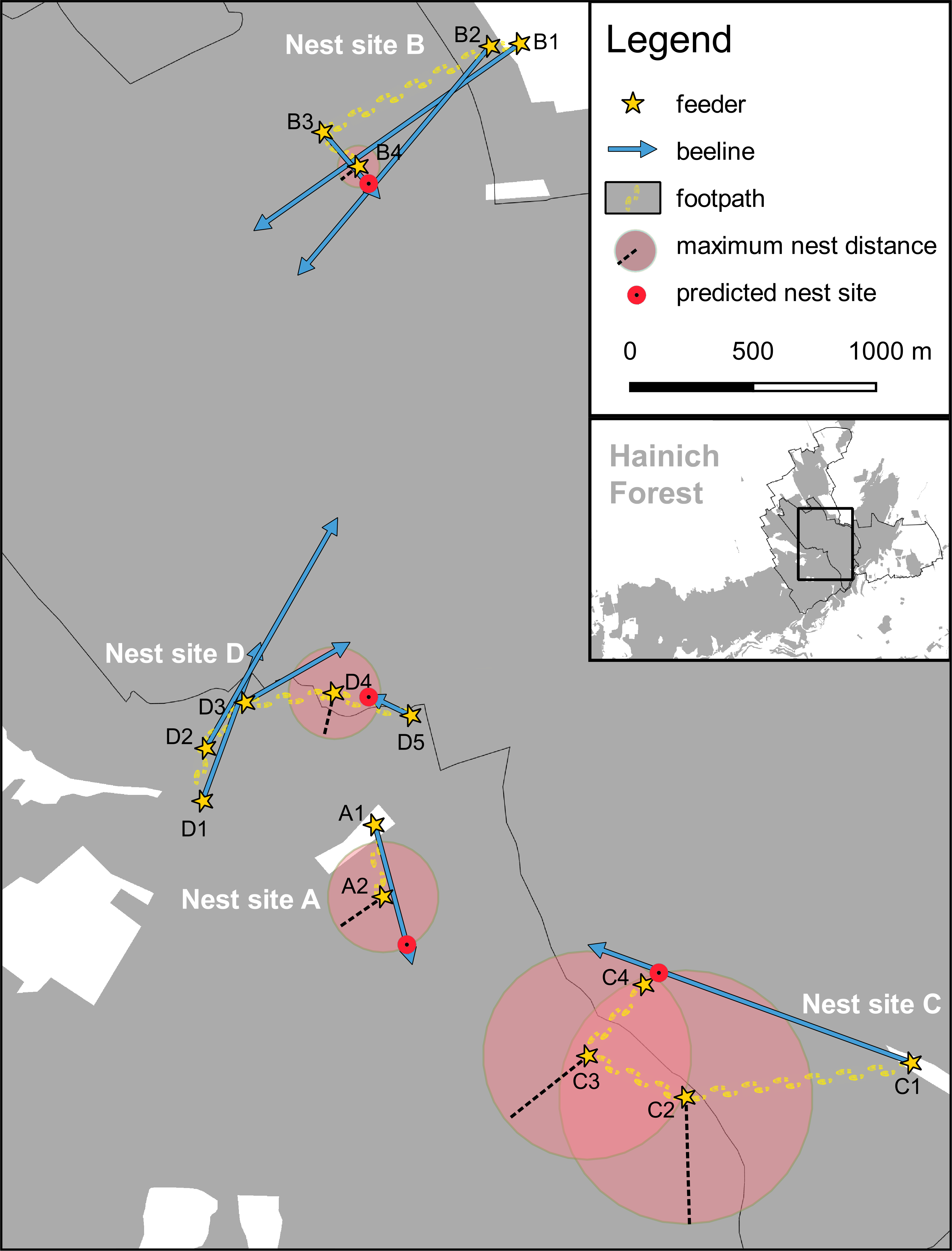

Supplement: Supplemental Information 2 — When we had problems observing vanishing bearings beneath the dense tree canopy, we used the minimum round trip time to calculate the maximum distance from the feeder to the nest (red transparent circles). Nest site A was predicted to be on the beeline from feeder A1 and inside the maximum nest range obtained at feeder A2. Nest site B was predicted to be at the crossing of the beelines from the feeders B2 and B3, and within the maximum nest range inferred from feeder B4. Nest site C was predicted to be on the beeline from feeder C1 and very close to the feeder C4 where the round trip time was shortest (Note that the predicted nest site for C lies also within the maximum nest ranges inferred from the feeders C2 and C3). Nest site D was predicted based on the beeline from feeder D5 and the maximum nest range inferred from feeder D4 (Map data © Mapbox, © OpenStreetMap). [file peerj-06-4602-s002.png]

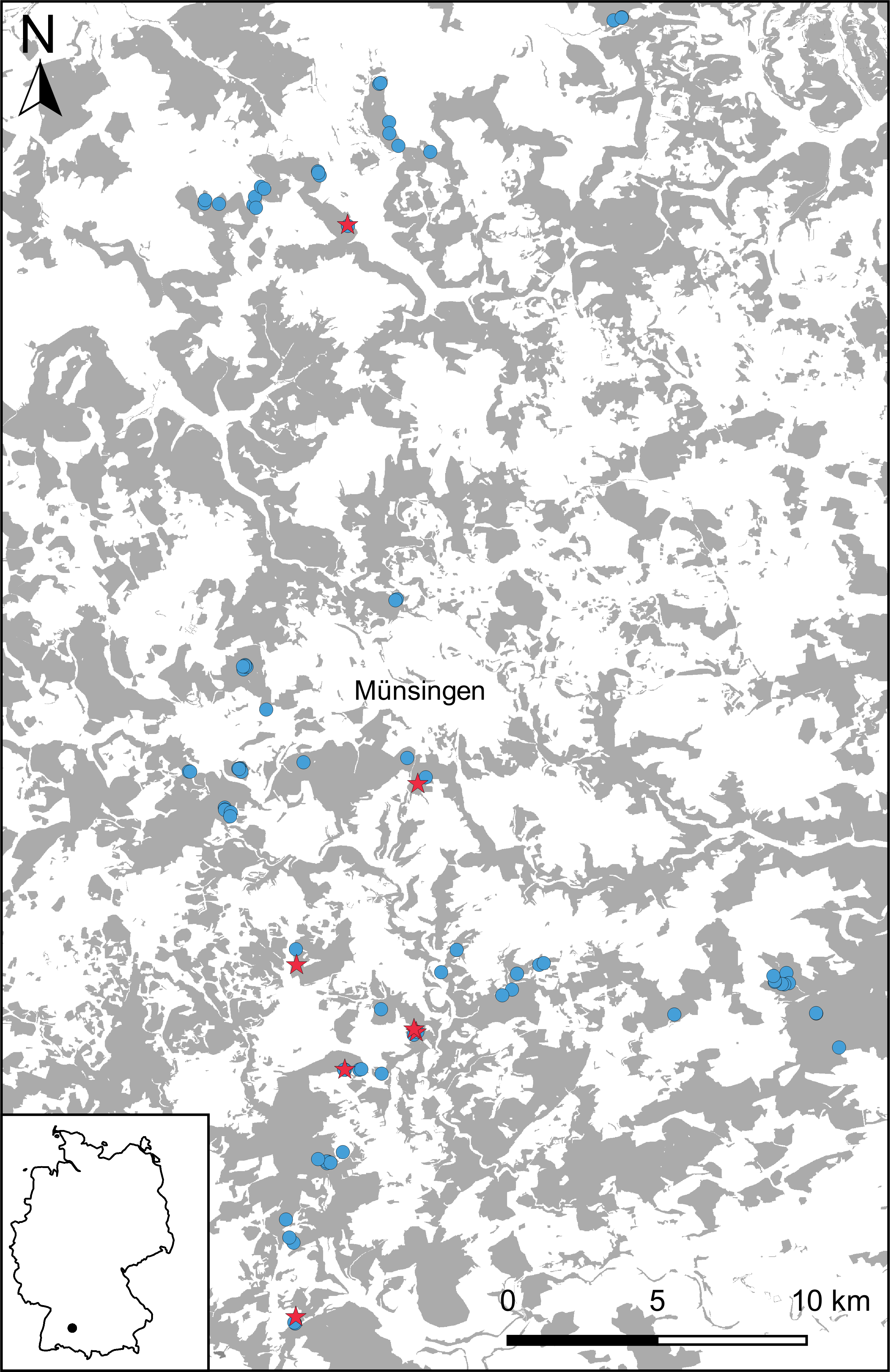

Supplement: Supplemental Information 3 — Map of the 98 inspected cavity trees (blue dots) in the Biosphere Reserve Swabian Alb with the 7 cavity trees occupied by honey bees (red asterisks) (Map data © Mapbox, © OpenStreetMap). [file peerj-06-4602-s003.png]
